# Analysis of fetal MRI data reveals no effect on liver maturation following prenatal alcohol exposure

**DOI:** 10.1007/s00330-026-12461-3

**Published:** 2026-03-16

**Authors:** Michael Schwarz, Martin Watzenboeck, Marlene Stuempflen, Katharina Tontsch, Patric Kienast, Julia Binder, Tim Dorittke, Rebecca Sokol, Felix Ragnar Merlin Koenig, Caroline Schwarz, Thomas Reiberger, Daniela Prayer, Gregor Kasprian, Victor Ulrich Schmidbauer

**Affiliations:** 1 Division of Gastroenterology and Hepatology, Department of Internal Medicine III, Medical University of Vienna, Vienna, Austria; 2 Department of Gastroenterology and Hepatology, University Hospital Sankt Pölten, Pölten, Austria; 3 Department of Biomedical Imaging and Image-guided Therapy, Medical University of Vienna, Vienna, Austria; 4 Division of Obstetrics and Feto-Maternal Medicine, Department of Obstetrics and Gynecology, Medical University of Vienna, Vienna, Austria

**Keywords:** Fetal MRI, Prenatal alcohol exposure, Liver, Fetal alcohol spectrum disorders, Radiomics

## Abstract

**Objective:**

Prenatal alcohol exposure (PAE) of the fetus may result in physiognomic alterations and/or impediment of cognitive development, summarized as fetal alcohol spectrum disorders (FASDs). Although alcohol is metabolized in the liver, the effects of PAE on the fetal liver are unknown.

**Materials and methods:**

For this study, *n* = 21 prenatal MRIs of fetuses with PAE and *n* = 45 MRIs of fetuses without PAE were included. To assess macroscopic organ development, liver volumetry was conducted and compared to an established prenatal estimation formula. A texture-based radiomics analysis based on ensemble (random forest) and linear (L2-penalized logistic regression) models was conducted.

**Results:**

PAE and non-PAE fetuses were age-matched (gestational age 27.4 [IQR 25.1–30.0] vs. 26.7 [25.4–29.3] weeks, *p* = 0.650). PAE had no effect on liver volume (normalized per gestational week, *p* = 0.971) or signal intensity (SI) in T1 (*p* = 0.574) and T2 (*p* = 0.104). MRI-derived liver volumetry correlated strongly with a gestational age-based volume estimation regardless of PAE (both r > 0.75, *p* < 0.001). A texture-based radiomics analysis was unable to discern between PAE and no-PAE fetuses (ROC-AUC = 0.43, 95% CI 0.29–0.57 for random forest, ROC-AUC = 0.40, 95% CI 0.31–0.60 for L2-penalized logistic regression).

**Conclusion:**

PAE had no significant effect on signal intensity of the fetal liver, and gestational-age-adjusted liver volumes did not differ between fetuses with and without PAE, suggesting no relevant effect on organ development. Texture-based radiomics were unable to identify PAE. Hence, alterations of the liver in fetal MRI with known PAE warrant investigations for causes other than alcohol.

**Key Points:**

***Question*** *Prenatal alcohol exposure can lead to morphological and neurocognitive changes in the fetus. The effect on the fetal liver is unknown.*

***Findings*** *No differences in fetal liver volume or signal intensity were found regarding alcohol exposure. Texture-based radiomics analysis was unable to discern between the two groups.*

***Clinical relevance*** *Fetal MRI and radiomics analysis were unable to detect effects of alcohol exposure on the fetal liver. Aberrations of the fetal liver on MRI in patients with a history of prenatal alcohol exposure warrant investigation for other causes.*

**Graphical Abstract:**

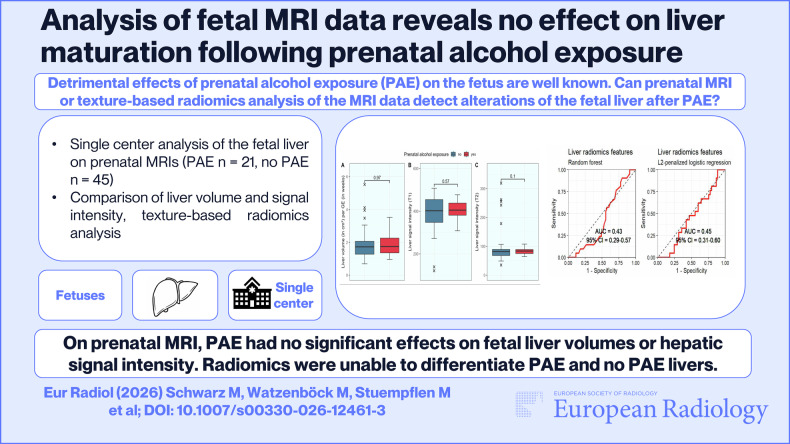

## Introduction

The detrimental effects of maternal alcohol consumption during pregnancy on the unborn child were first described more than 50 years ago, and the observed specific physiognomic characteristics were defined as fetal alcohol syndrome (FAS) [1, 2]. Today, prenatal alcohol exposure (PAE) is the recognized cause for FAS, but also for behavioral and cognitive disorders or delays in development in the absence of physical changes, which is summarized as fetal alcohol spectrum disorders (FASD) [3, 4]. Approximately 10% of pregnant women drink alcohol during pregnancy, while in the European region, 25% of pregnant women consume some degree of alcohol during pregnancy [5]. Concordantly, the prevalence of FASD is the highest in Europe, with 2% of the general population, while the global average is 0.7% [5]. One in 13 women who consume alcohol during pregnancy will give birth to a child with FASD, resulting in about 630,000 children born with FASD globally every year [6], thus making FASD the most common of preventable conditions associated with birth defects and delays or limitations in cognitive development [7]. The alcohol-induced somatic changes of FASDs are detectable via ultrasound as soon as the second trimester [8]. Studies found no safe level of alcohol consumption during pregnancy, and complete abstinence during pregnancy or cessation as soon as possible is recommended [9]. The alcohol-induced somatic changes seen in FASDs are detectable via ultrasound as soon as the second trimester [8]. While alcohol is metabolized in the liver, the effects of PAE on the fetal liver are not known.

Recent years have seen an exponential growth of imaging modalities, post-processing measurements, and new ways to assess the obtained data. Radiomics converts radiological images into datasets and allows a quantitative assessment as well as the application of machine learning models, thereby potentially unveiling findings inaccessible to the human eye [10, 11]. Application in the field of hepatic imaging showed that magnetic resonance imaging (MRI)-based radiomics may allow a better liver fibrosis assessment than current non-invasive tests like FIB4 or aspartate aminotransferase–to–platelet ratio index (APRI) [12] and the differentiation between multiple etiologies of chronic liver disease [13, 14]. Fetal MRI is a prenatal imaging technique that allows an extensive and accurate analysis of the fetal organ systems, including the liver [15]. It has been shown that PAE leads to alteration of the fetal brain [16], but data regarding the effect of PAE on fetal liver imaging are sparse. Furthermore, combining fetal MRI and radiomics has shown robustness to predict prenatal lung development, the risk for disorders of the placenta, or adverse outcome in neurological development post partum [17–19]. As of today, no radiomics data regarding the effect of alcohol on the fetal liver exists; however, PAE has been associated with increased birthweight and metabolic syndrome later in life, both risk factors for the development of steatotic liver disease [20, 21].

The aim of this investigation was to obtain insights into the effects of PAE on the fetal liver and potential maturation aberrancies by assessing hepatic signal intensities and MRI-based volumetry, as well as converting the obtained imaging data into radiomics for a texture-based analysis to identify changes beyond human perception.

## Methods

### Patients

Pregnant women with singleton pregnancies undergoing fetal MRI at a tertiary care center in Vienna, Austria, from November 2018 until April 2021 were included. The patients were referred by prenatal ultrasound centers, where the indication for prenatal MRI was made. At baseline, maternal medical history and gestational age (in weeks and days post menstruation) were assessed. To detect and quantify PAE, two standardized questionnaires (PRAMS: Pregnancy Risk Assessment Monitoring System, T-ACE questions: Tolerance, Annoyance, Cutting-down, Eye-Opener) were used [22, 23]. According to self-reported alcohol use, the patient cohort was dichotomized into patients with “no PAE” and any PAE. “Binge drinking” was defined as any event with 4 or more standard drinks during pregnancy. For a sub-analysis, patients were also divided into “binge drinking” and “no PAE.” Patients with and without PAE were gestational age-matched in a 1:2 ratio. Fetuses with apparent liver defects were excluded. When available, post-partum laboratory and/or imaging data were assessed.

### Fetal MRI

Fetal MRIs were performed according to the international clinical practice guideline [15]. Images were acquired with the mother in a supine position and within 30 min. No contrast media or sedation was used. MRI scans were conducted using 1.5-Tesla (Philips Ingenia/Intera) and 3.0-Tesla magnets (Philips Achieva). Imaging included T1- and T2-weighted sequences in three orthogonal planes (slice thickness 2.0–4.5 mm, echo time 100–140 ms, field of view 200–230 mm, in-plane resolution 0.62/0.62–1.17/1.17 mm) of the fetal liver. Fetal T1- and T2-based signal intensity measurements were obtained on Picture Archiving and Communication System (PACS) workstations. For organ volumetry and radiomics, fetal livers were segmented for each patient individually by a physician via ITK-SNAP 4.0.1 (Penn Image Computing and Science Laboratory (PICSL)) on T2-weighted imaging data.

### Statistics

Statistical analysis and data visualization were done in RStudio, Version 2024.04.2, Build 764 (Posit Software PBC). Data visualization was done through the R-package “ggplot2,” version 3.5.1. Variables are presented as absolutes (and percent) or mean and standard deviation (± SD). Unpaired *t*-test was used to compare groups. Correlations were calculated using Pearson’s correlation coefficient. MRI-based organ volumetry was compared to gestational age-based fetal liver volume estimation according to the formula postulated by Laudy et al [24]: = −122.22 + 6.07 × (gestational age in weeks). The level of significance was set at a *p*-value of 0.05 and the level of confidence at 95%.

### Radiomics

Liver segmentation masks and corresponding MRI scans were exported in NIfTI format. Radiomics feature extraction was carried out using the open-source Python package *pyradiomics* (version 3.1.0), executed within a Python 3.9.21 environment [25]. Image normalization was performed by enabling the *normalize* parameter and setting *normalizeScale* to 100. To avoid negative gray values for voxels below the mean during normalization, the *voxelArrayShift* parameter was set to 300—equivalent to 3 standard deviations multiplied by the scaling factor—ensuring that only extreme outliers (more than 3 SDs below the mean) would remain negative. Image discretization was applied using a *binWidth* of 5. For image resampling, the interpolator was set to *sitkBSpline*, and the voxel spacing was standardized using the *resampledPixelSpacing* parameter set to [2, 2, 2]. The extracted radiomics features encompassed the following classes: First Order (*n* = 18), 3D Shape (*n* = 14), Gray Level Co-occurrence Matrix (GLCM, *n* = 22), Gray Level Size Zone Matrix (GLSZM, *n* = 16), Gray Level Run Length Matrix (GLRLM, *n* = 16), and Gray Level Dependence Matrix (GLDM, *n* = 14).

### Radiomics-based classification of PAE

Random forest and L2-penalized logistic regression classifiers, as implemented in the open-source Python package scikit-learn (version 1.5.1), were trained to predict PAE based on fetal MRI liver radiomics features. In addition to radiomics features, gestational age was included as a feature in the training data to control for differences in gestational age. To evaluate model performance, a stratified 20-fold cross-validation scheme was employed. Stratification was used to compensate for class imbalance, since patients with and without PAE were gestational age-matched in a 1:2 ratio. Classifiers were iteratively trained on 95% of the dataset and evaluated on the remaining 5%, which was repeated 20 times. The predicted probabilities for each class were saved and used for ROC curve construction. When using logistic regression classifiers, features were standardized by removing the mean and scaling to unit variance. Importantly, standardization parameters were derived exclusively from the training partition during each cross-validation fold, to prevent data leakage into the testing partition.

### Ethics

This study was conducted in concordance with the principles of the Declaration of Helsinki, as approved by the local ethics committee. Informed consent for the scientific use of the acquired data was obtained prior to MRI.

## Results

We included *n* = 21 prenatal MRIs of patients with and *n* = 45 prenatal MRIs of patients without alcohol exposure during pregnancy. Two patients with PAE had repeated MRIs at gestational weeks 22 and 28, and 22 and 29, respectively. Median maternal age, gestational age, and fetal sex did not differ significantly between the two groups. In the PAE cohort, the majority reported weekly alcohol consumption of *n* = 1 standard alcoholic beverage per week (*n* = 14, 66.7%). Singular binge drinking episodes, defined as episodes of 4 or more, were reported by *n* = 4 (19.0%) patients in the PAE cohort. See Table 1. For patient details and indications for fetal MRI, see Supplementary Table 1.

**Table 1:** Patient characteristics dichotomized by prenatal alcohol exposure (PAE)

	No PAE	PAE	*p*-value
	*n* = 45	*n* = 21	
Maternal age in years, mean (± SD)	31.2 (± 4.9)	29.5 (± 4.7)	0.245
Gestational age in weeks, mean (± SD)	27.9 (± 4.2)	27.5 (± 3.9)	0.702
Fetal male sex, *n* (%)	28 (62.2%)	16 (76.2%)	0.400
Standard drinks per week			
0	45 (100%)	0 (0%)	
1	0 (0%)	14 (66.7%)	< 0.001
2	0 (0%)	3 (14.3%)	
3	0 (0%)	2 (9.52%)	
4 or more	0 (0%)	2 (9.52%)	
Binge drinking episode (≥ 4 drinks/day)	0 (0%)	4 (19.0%)	0.002

Fetal liver volumes as assessed through MRI-based volumetry showed smaller liver volumes in the PAE cohort compared to the no-PAE cohort, although not statistically significant (mean liver volume PAE 53.87 ± 26.79 [95% confidence interval (CI) 41.67–66.06] cm³ vs. no-PAE 55.46 ± 35.90 [CI 44.67–66.24] cm³, *p* = 0.874). Normalization of fetal liver volume to gestational week also showed no significant difference in organ size (PAE 1.88 [CI 1.57–2.19] cm³/week vs. no-PAE 1.89 [CI 1.61–2.17] cm³/week, *p* = 0.971). When dichotomizing the cohort into patients who reported episodes of binge drinking during pregnancy and no PAE, the difference between fetal liver volume was more pronounced (PAE 37.91 [CI 24.16–51.65] cm³ vs. no-PAE 55.46 [44.67–66.24] cm³, *p* = 0.028), but did not attain statistical significance when normalized to gestational age (1.47 ± 0.33 [CI 1.07–1.88] cm³/week vs. no-PAE 1.89 ± 0.94 [CI 1.61–2.17] cm³/week, *p* = 0.059).

**Fig. 1:**
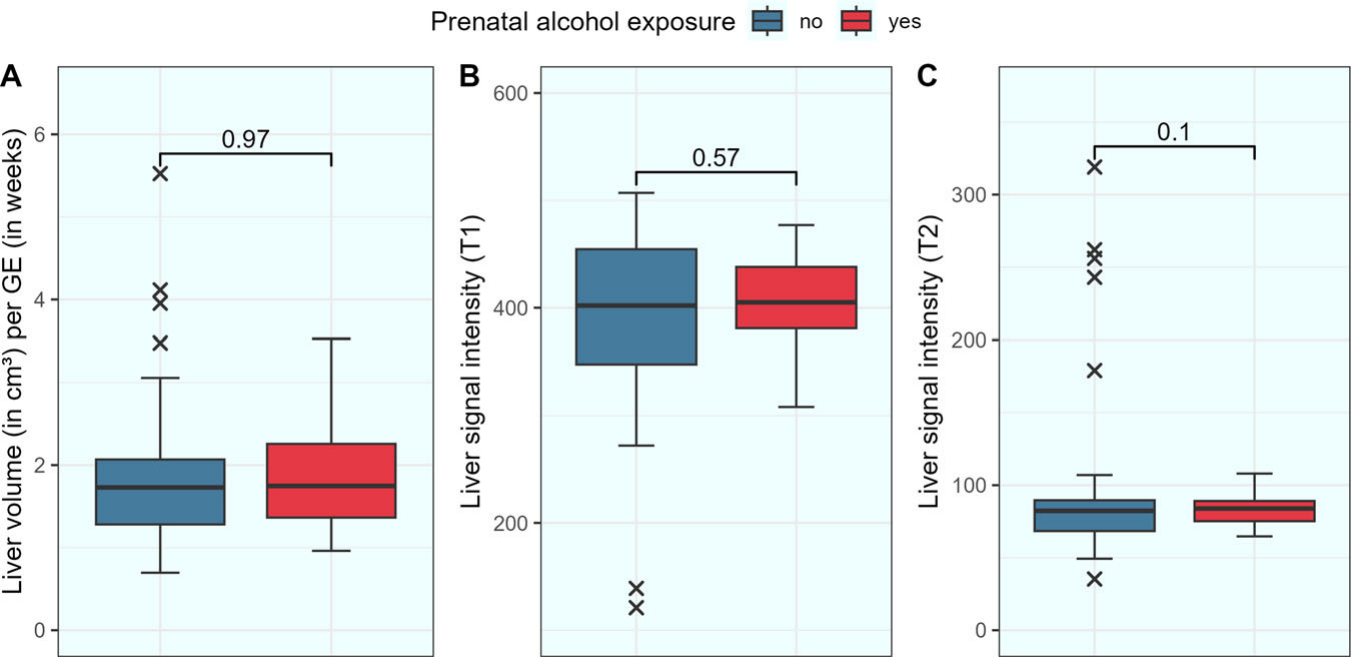
Effects of prenatal alcohol exposure on magnetic resonance imaging findings. Prenatal alcohol exposure resulted in no statistically significant effect on imaging-derived fetal liver volumes (normalized per gestational age (GE) in weeks (**A**) or hepatic signal intensities in T1- (**B**) or T2-weighted (**C**) imaging)

T1- and T2-weighted imaging signal intensity of the fetal liver did not differ irrespective of PAE (Fig. 1; mean T1 SI: PAE 401.52 ± 50.70 [CI 378.45–424.60] vs. no-PAE 391.51 ± 86.45 [CI 363.49–419.54], *p* = 0.574; mean T2 SI: PAE 82.17 ± 11.64 [CI 76.87–87.46] vs. no-PAE 97.31 ± 59.01 [79.58–115.04], *p* = 0.104). Dichotomization into solely patients who reported episodes of binge drinking during pregnancy and patients without alcohol exposure showed similar results. See Table 2.

**Table 2:** Comparison of radiological findings between fetuses with and without prenatal alcohol exposure (PAE)

	No PAE	PAE	*p*-value
	*n* = 45	*n* = 21	
Liver volume in cm³, mean (± SD)	55.46 (± 35.90)	53.87 (± 26.79)	0.842
Liver volume in cm³ per gestational week, mean (± SD)	1.89 (± 0.94)	1.88 (± 0.69)	0.971
Liver T1 signal intensity	391.51 (± 86.45)	401.52 (± 50.70)	0.574
Liver T2 signal intensity	97.31 (± 59.01)	82.17 (± 11.64)	0.104

	No PAE	Binge drinking	*p-*value
	*n* = 45	*n* = 5	
Liver volume in cm³, mean (± SD)	55.46 (± 35.9)	37.91 (± 11.07)	0.028
Liver volume in cm³ per gestational week, mean (± SD)	1.89 (± 0.94)	1.47 (± 0.33)	0.059
Liver T1 signal intensity	391.51 (± 86.45)	375.40 (± 60.15)	0.612
Liver T2 signal intensity	97.31 (± 59.01)	88.20 (± 15.96)	0.430

No differences in organ size or signal intensity were found in fetuses with and without PAE on magnetic resonance imaging. Comparing patients who reported episodes of binge drinking during pregnancy (defined as days with more than 4 standard drinks) and patients without PAE suggested a (not statistically significant) reduction in liver volume in fetuses with binge drinking exposure

**Fig. 2:**
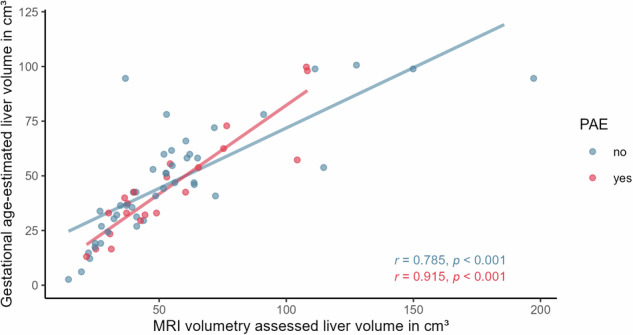
Comparison of estimated and actual fetal liver volume. Gestational age-based estimation of the fetal liver volume showed an excellent correlation with the actual liver volume. Prenatal alcohol exposure (PAE) had no discernable effect on liver maturation

**Fig. 3:**
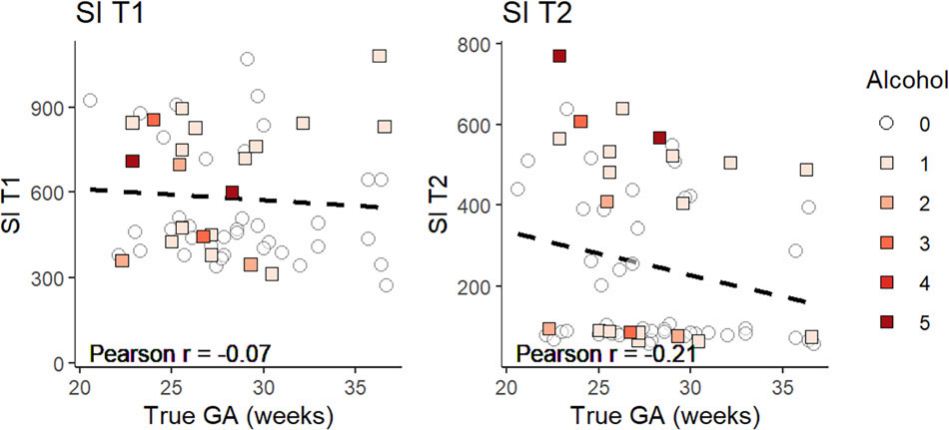
Conventional analysis based on signal intensity (SI), gestational age (GA), and prenatal alcohol exposure (in standard drinks per week). T1 and T2 signal intensity did not correlate with gestational age.

To assess the effect of PAE on the development of the fetal liver, a gestational age-based formula for the estimation of the fetal liver volume was used [24]. A strong correlation between the estimated and the actual liver volume was observed (mean MRI-based liver volumetry 54.95 ± 33.07 [CI 46.82–63.08] cm³ vs. mean gestational-age-based formula predicted volume 46.53 ± 24.62 [CI 40.48–52.58] cm³, Pearson’s r = 0.812 [CI 0.71–0.88], *p* < 0.001). The correlation remained strong when dividing the cohort according to alcohol exposure (PAE MRI volumetry-based liver volume vs. estimation formula-based volume r = 0.915 [CI 0.79–0.97], *p* < 0.001; no-PAE MRI liver volume vs. estimation formula r = 0.785 [CI 0.64–0.88], *p* < 0.001). Furthermore, when assessing signal intensity, gestational age, and PAE, no trend regarding the distribution of fetuses with PAE was observed. Hence, no significant effect of alcohol exposure on hepatic maturation was evident. See Figs. 2 and 3.

Post-partum clinical data were available in four newborns with PAE. One fetus of a mother with reported binge drinking during pregnancy had elevated levels of gamma-glutamyl-transferase (gGT, 143 U/L) and aspartate transaminase (AST, 49 U/L). gGT declined over the following days, and the abdominal ultrasound was unremarkable. Elevated gGT was also seen in another newborn with PAE (129 U/L), which resolved over time. Again, the abdominal ultrasound was unremarkable. A third fetus with PAE showed a delayed and prolonged rise in gGT from post-partum day 11–25 (maximum gGT 695 U/L). Data regarding abdominal ultrasound were not available. See Supplementary Fig. 2.

To determine whether radiomics (e.g., texture or shape-based features) could differentiate between patients with and without PAE based on fetal MRI liver imaging, ensemble (random forest) and linear (L2-penalized logistic regression) models were trained to retroactively predict PAE based on fetal liver MRI radiomics features. Importantly, neither of the two models could discriminate between patients with or without PAE (ROC-AUC = 0.43, 95% CI 0.29–0.57 for random forest, ROC-AUC = 0.40, 95% CI 0.31–0.60 for L2-penalized logistic regression).

## Discussion

In this manuscript, we report on a well-characterized, gestational age-matched cohort of in total *n* = 66 prenatal MRI reports for different indications, where one third of the population reported PAE. Fetal liver volumes did not differ between fetuses with and without PAE—even when adjusted for gestational age. Also, signal intensities of the fetal liver did not differ between the two groups. To identify miniscule alterations of the liver, a texture-based Radiomics classification model was created to potentially identify PAE-related changes. However, the designed model could not differentiate between patients with or without PAE. Hence, we assume that PAE causes no relevant effect on the fetal hepatic parenchyma or organ maturation detectable by classical MRI and/or radiomics.

Although there is no safe level of alcohol consumption during pregnancy, prenatal alcohol exposure is still a common risk factor for fetal developmental delay—especially in Europe, where a PAE rate of 25% was reported in some countries [5, 9]. In the United States of America and in Europe, the prevalence of FASD is high, with an estimated 2–5 fetuses per 100 births [4, 26]. While the role of PAE in the development of FASD is well established and fetal MRI is capable of detecting alterations of the fetal brain after alcohol exposure [16, 27, 28], its effect on development of the fetal liver is still unclear. PAE has been associated with low weight, insulin resistance, growth retardation after birth, and the development of diabetes mellitus later in life [20, 29, 30]. Together, these factors may further contribute to metabolic dysfunction-associated steatotic liver disease (MASLD) in the adolescent and adult patient [21, 31]. Furthermore, an association of PAE and the development of some childhood cancers like neuroblastoma, leukemia, and brain tumors has been described [32, 33]. While the role of PAE in the development of FASD is well established and fetal MRI is capable of detecting alterations of the fetal brain after alcohol exposure [16, 27, 28], data regarding the effect of PAE on the fetal liver are lacking. Besides superficial description of changes in imaging modalities, radiomics might allow a deeper assessment and quantification of imaging data and detection of alterations otherwise inaccessible to the human eye [11]. Radiomics has already been applied in fetal MRI of the lung, where a radiomics-based approach allowed evaluation of lung maturity [17] or in fetal MRI and radiomics-based assessment of the placenta to predict fetal growth restriction [34]. Regarding liver conditions, application of radiomics on MRI-derived data allows the identification of alcohol exposure in adult humans and rats [35]. Radiomics also allows assessment of liver fibrosis and an estimation of etiology in adult patients [12, 13], but data regarding its use in fetal liver MRI are lacking.

**Fig. 4:**
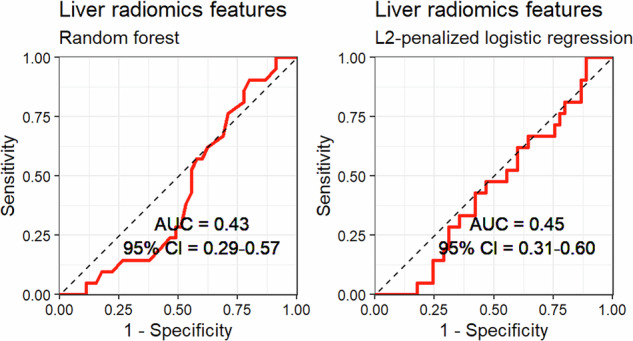
Classification of prenatal alcohol exposure status based on fetal liver magnetic resonance imaging radiomics features. Based on liver MRI radiomics features, neither random forest nor L2-penalized logistic regression models were able to discriminate between fetuses with and without prenatal alcohol exposure

In our study, no significant effect of PAE on the fetal liver was discernable in radiological analysis, as well as via texture-based radiomics analysis of the extracted imaging data. The observed AUC values of the random forest or logistic regression models were both slightly below 0.5 (0.43 and 0.45, respectively, Fig. 4), which might be suggestive of inverse or confounding signals in the dataset. Data acquisition was performed carefully, and no potentially affecting covariates were identified. Furthermore, for both models, the confidence intervals spanned across 0.5. Hence, we assume that the AUCs below 0.5 are mere chance and not due to a relevant bias in the dataset. While it has been shown in adults that microstructural remodeling in the liver often produces meso-/macro-scale imaging correlates—i.e., changes in texture, intensity distributions, and spatial heterogeneity—that radiomics can quantify, based on our data, we cannot exclude that PAE produces metabolic changes to the liver that cannot be captured by radiomics [36–38]. Further research, in particular on laboratory parameters of newborns with PAE and/or post-mortem studies, might help to elucidate the potential mechanisms.

While no macroscopic alterations of the fetal liver were observed in the PAE cohort, the detrimental effects of alcohol exposure during pregnancy are well-known, and the World Health Organization recommends complete cessation of alcohol consumption during pregnancy as no safe level is known [39, 40]. Children with FAS exhibit craniofacial (thin upper lip, smooth philtrum, short palpebral fissure) and cerebral alterations (decreases in gray matter thickness, volume reduction, decreased cortical gyrification) post partum [4]. The individual indications for fetal MRI in our cohort are presented in Supplementary Table 1, and while the cohort size is relatively small and the reported outcomes heterogeneous, alterations of the head and face (microcephaly, midface hypoplasia, exophthalmos, cleft lip and palate) were exclusive to the PAE cohort. Furthermore, during pregnancy, the placenta assumes metabolic pathways usually domains of the liver, and thus, potentially sparing the fetal liver of the detrimental effects of alcohol, which might explain the absence of detectable hepatic alterations [41, 42]. In line with this, placental aberrations have been described in mothers with alcohol consumption during pregnancy [16, 43, and Stuempflen et al (2026), unpublished]. In our cohort, alterations of the placenta were exclusive to the PAE cohort as seen in Supplementary Table 2.

Despite the absence of structural MRI correlates, postnatal biochemical alterations were observed in some PAE-exposed newborns, suggesting that metabolic changes—physiological or pathological—may precede or occur independently of macrostructural changes detectable by current MRI techniques. Post-partum laboratory data were available in four patients of the PAE cohort. Transaminases, like aspartate transaminase (AST) and alanine aminotransferase (ALT), are enzymes inside the hepatocytes’ cytoplasm or mitochondria, and elevated serum levels are indications of hepatic inflammation and/or necrosis [44]. Although broadly available biomarkers for liver damage, no universally accepted normal range for AST or ALT has been established in adult patients due to globally varying kits and cut-offs; however, 40 U/L is commonly referred to as “upper limit of normal” [45–47]. Similar levels have been described in newborns, with usually AST higher than ALT and a mild effect of alcohol consumption on AST levels [48, 49]. In our cohort, despite no evident changes on MRI or MRI-based radiomics, three out of four fetuses with available post-partum laboratory data had transaminases above 40 U/L. Elevated AST/ALT values tended to decline spontaneously after birth. Another hepatic enzyme, the gamma-Glutamyl-Transferase (gGT), was also elevated above the level of adult patients in three out of four fetuses. gGT is associated with alcohol consumption in adults; however, also associated with the induction of hepatic activity in the newborn, and increasing levels after birth are expected [50]. One fetus of a mother with reported binge drinking had elevated gGT at birth, which declined in the following days. Alkaline phosphatase (ALP), a hepatic enzyme associated with bile flow and cholestasis, was slightly elevated in one fetus of a mother who reported binge drinking episodes. However, ALP is also involved in bone metabolism, and high blood levels can be expected in newborns [51]. See Supplementary Fig. 2.

Our study has limitations we want to address. As fetal MRI is not a widely available procedure and standardized assessment of alcohol consumption are rarely available with imaging reports, the sample size is relatively small. However, the cohort and sub-cohorts were homogeneous without apparent differences in relevant characteristics. The confidence intervals of the compared groups were predominantly overlapping, supporting the robustness of the data and the described lack of observable biological effect on the fetal liver on MRI. Although alcohol consumption was assessed by standardized questionnaires, the data were self-reported. No other methods to evaluate alcohol consumption, like blood-based tests, were used. The small sample size is especially apparent in the small sub-group of mothers who reported binge drinking episodes during pregnancy (*n* = 5 of *n* = 21 mothers with alcohol exposure). In this sub-group, we found smaller organ volumes of fetal livers compared to fetuses without PAE; however, the finding did not maintain its statistical significance when adjusted for gestational age. This advises for careful interpretation as the effect might be unstable due to the small sub-sample size and highlights the need for further investigation in larger cohorts, as despite the negative data in our manuscript, an effect from high-level exposure could exist. While quantitative MRI approaches might be superior in depicting the biochemical and microstructural processes of liver injury, image acquisition times and fetal motion limit their use in fetal MRI in vivo, and they are not part of standardized fetal MRI acquisition protocols [52, 53]. Post-partum laboratory data were not available in all patients, as some patients were external referrals and follow-up was conducted at a different center. Stuempflen et al assessed PAE-associated alterations of the fetal brain in an overlapping cohort and found significant changes to the corpus callosum and periventricular zone in alcohol-exposed fetuses [16]. The reported effects are potential forebearers of postnatal delays in neuro-development, compatible with FASD [54]. In our cohort, brain alterations detectable on fetal MRI were exclusive to the PAE cohort and are reported in Supplementary Table 2.

In summary, fetal MRI is capable of a detailed assessment of fetal organs that complements ultrasound in many ways. Transferring these imaging reports into radiomics data may allow an even more detailed and deeper analysis. In our manuscript, no direct effect of alcohol exposure on fetal liver maturation or MR-morphology was detectable. Deeper analysis through a radiomics-based approach did not identify any potentially alcohol-related changes in the fetal liver. For the clinical radiologist and obstetrician/gynecologist, alterations of liver morphology on imaging studies should prompt the search for differential diagnoses other than alcohol-related complications.

## Supplementary information

ELECTRONIC SUPPLEMENTARY MATERIAL

## Data Availability

As the data for this manuscript contains sensitive patient information, it is not publicly available. Data generated or analyzed during the study is potentially available from the corresponding author upon reasonable request and in anonymized form.

## Abbreviations

ALP: Alkaline phosphatase
AST: Aspartate transaminase
CI: Confidence interval
FAS: Fetal alcohol syndrome
FASD: Fetal alcohol spectrum disorders
FIB4: Fibrosis-4 index
gGT: Gamma-glutamyl-transferase
MRI: Magnetic resonance imaging
PAE: Prenatal alcohol exposure
ROC-AUC: Receiver operating characteristic area under the curve
