# Supplementary material for: Analysis of fetal MRI data reveals no effect on liver maturation following prenatal alcohol exposure
Source: Eur Radiol. 2026 Mar 16;36(8):6410–8. doi: 10.1007/s00330-026-12461-3 (PMC13341938; doi:10.1007/s00330-026-12461-3)
Supplement: Supplementary file 1 — ELECTRONIC SUPPLEMENTARY MATERIAL [file 330_2026_12461_MOESM1_ESM.pdf]

Analysis of fetal MRI data reveals no effect on liver maturation following prenatal alcohol exposure

ELECTRONIC SUPPLEMENTARY MATERIAL

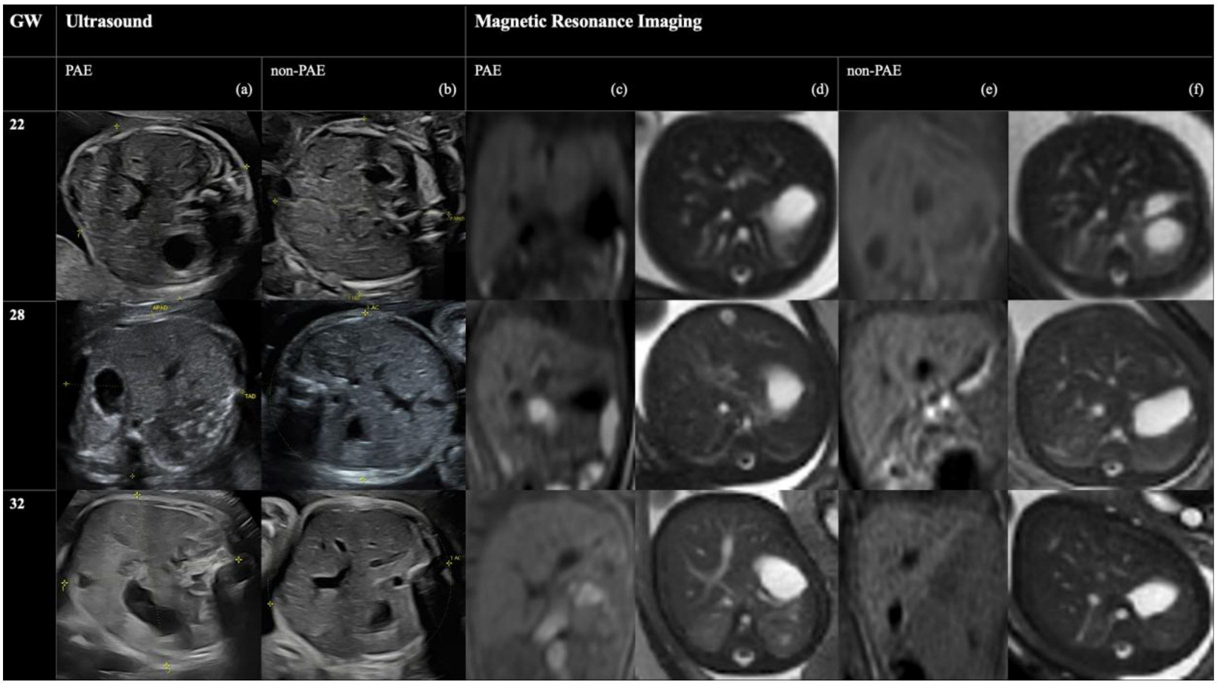

Supplementary Figure 1: Comparison of fetal liver imaging by ultrasound and magnetic resonance imaging in fetuses with and without prenatal alcohol exposure (PAE). Images were taken in different gestational weeks (GW, rows). On ultrasound imaging, no apparent differences regarding the fetal liver were found on between fetuses with (a) and without PAE (b). Similarly, no changes in signal intensity or organ morphology of the fetal liver in regards to PAE were found on T1- (c, e) and T2-weighted (d, f) magnetic resonance imaging.

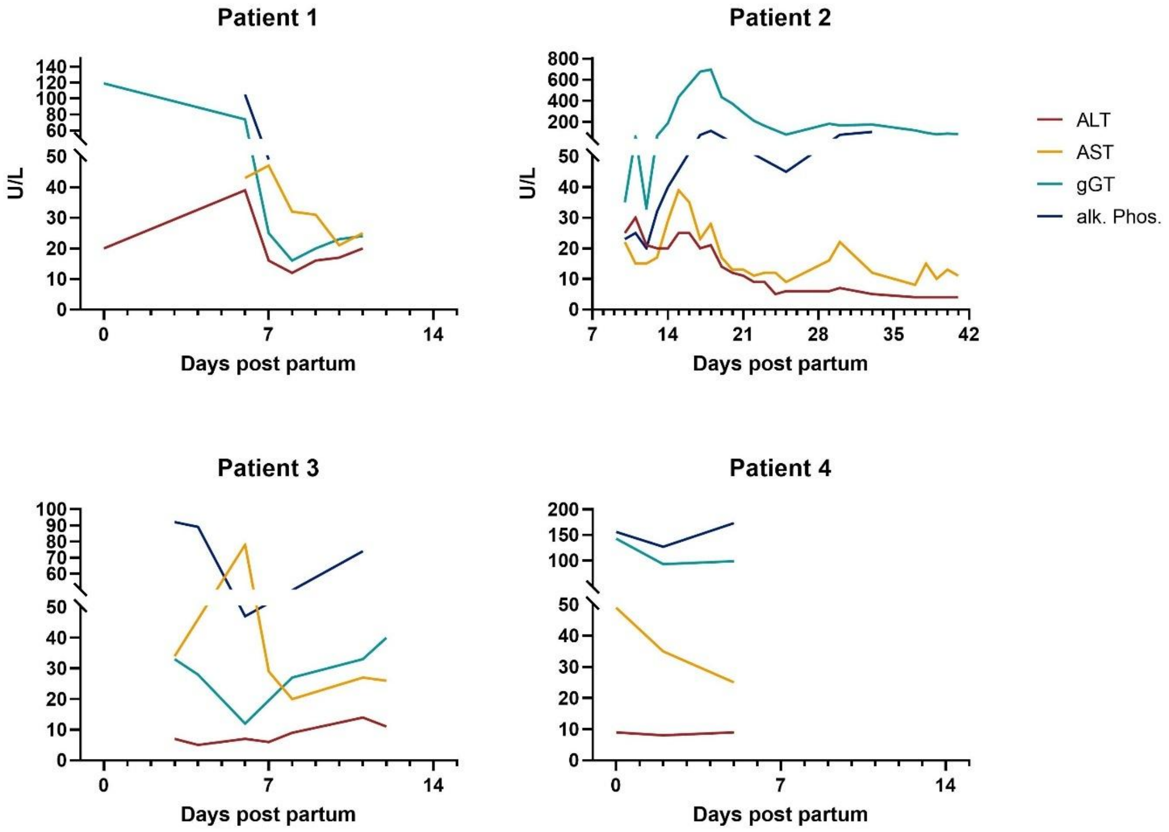

Supplementary Figure 2: Post-partum laboratory data of four fetuses with prenatal alcohol exposure. Markers of hepatic inflammation (AST, ALT) and cholestasis (gamma-GT, alkaline phosphatase) were commonly elevated. The mother of fetus #4 reported binge drinking during pregnancy. Post-partum fetal abdominal ultrasound in fetus #4 showed an unremarkable report. Abdominal ultrasound was also done in fetus #1, which too showed no remarkable results – especially regarding the liver.

Mat. age	Fetal sex	Gest. age, weeks	PAE	BDE	Reason for referral or main diagnosis on MRI
No prenatal alcohol exposure (PAE) or binge drinking episodes (BDE), n=45 MRIs					
22	Female	36	No	No	Ovarian cyst left
23	Female	23	No	No	Suspected kidney cyst; ureterocele left
23	Female	29	No	No	Suspected microcephaly, no pathology found
24	Female	23	No	No	Caudalised left kidney
24	Male	27	No	No	Suspected macrocephaly, no pathology found
24	Male	35	No	No	Hernia
25	Male	28	No	No	Suspected liver alternation on US, no pathology found
25	Male	29	No	No	CCAM left
26	Female	35	No	No	Ovarian cyst right
27	Male	24	No	No	Abnormal foot positioning, no pathology found
28	Male	24	No	No	Suspected venous malformation, no pathology found
28	Male	25	No	No	Suspected ovarian cyst, found elongated gallbladder
29	Female	25	No	No	Right pelvic kidney
29	Male	29	No	No	CCAM
30	Male	28	No	No	Polyhydramnion
30	Male	29	No	No	Suspected premature rupture of membranes (PROM), no pathology
30	Male	30	No	No	Multicystic kidneys, oligohydramnion
30	Male	31	No	No	Jejunal atresia, microcolon
31	Male	21	No	No	Control after heterotaxia in previous pregnancy, no pathology
31	Male	22	No	No	Polycystic dysplastic left kidney
31	Male	26	No	No	Dysplastic left kidney
31	Female	27	No	No	Subpulmonal sequester
31	Female	33	No	No	Congenital cystic adenomatoid malformation (CCAM)
31	Female	36	No	No	Ovarian cyst
32	Male	25	No	No	PROM
32	Female	27	No	No	gallbladder agenesia
32	Male	30	No	No	Connective tissue disorder in previous pregnancy, no pathology
33	Male	23	No	No	Suspected hemorrhage in cisterna magna, no pathology
33	Female	26	No	No	Splenic cyst
33	Female	27	No	No	PROM
33	Male	30	No	No	Bilateral pelvic kidney
33	Female	36	No	No	Club feet
34	Male	24	No	No	Cleft right lip and palate, suspect positioning of right lower extremity
34	Male	25	No	No	Left kidney agenesis
34	Male	32	No	No	Susptected large cisterna magna, no pathology
35	Female	27	No	No	Suspected PROM, no pathology
35	Male	33	No	No	Suspected hypokinesia of lower extremities, no pathology
36	Male	22	No	No	Cystic structure cranial of left kidney
37	Female	26	No	No	Suspected bowl loop distension, no pathology
37	Male	28	No	No	No pathology
39	Female	20	No	No	Genetic disorder in previous pregnancy, no pathology found
40	Male	25	No	No	Suspected abdominal cyst, found elongated gallbladder
40	Female	26	No	No	Left hand missing
40	Male	26	No	No	PROM, lung hypoplasia
40	Male	27	No	No	Suspected macroglossia, no pathology
Prenatal alcohol exposure (PAE) and binge drinking episodes (BDE), n=21 MRIs					
19	Male	24	Yes	Yes	Suspected microcephaly, no pathology
20	Male	26	Yes	Yes	Alcohol exposure, no pathology found
23	Male	22	Yes	No	Venous malformation
24	Male	26	Yes	No	Gastroschisis
26	Female	25	Yes	No	Diaphragmal eventration, lung hypoplasia
26	Male	36	Yes	No	Mitral valve stenosis, aortic hypoplasia
26	Male	36	Yes	No	Aortic isthmus stenosis
27	Male	25	Yes	No	Right-sided aortic arch, upper lobe bronchial compression
29	Male	22*	Yes	No	Lymphangioma left leg/sacral
29	Male	29	Yes	No	Midface hypoplasia, anal atresia, exophthalmus

29	Male	29*	Yes	No	Lymphangioma left leg/sacral
30	Female	25	Yes	No	Right aortic arch
30	Male	25	Yes	Yes	Venous malformation
32	Male	27	Yes	No	Venous malformation
33	Female	22*	Yes	Yes	Alcohol use disorder, small head biometry
33	Female	28*	Yes	Yes	Alcohol use disorder, small head biometry
35	Male	27	Yes	No	Lissencephaly in previous child, no pathology
35	Female	32	Yes	No	Bilateral plexus cysts
36	Male	29	Yes	No	Club feet
37	Male	30	Yes	No	Polyhydramnion
41	Male	25	Yes	No	Cleft lip and palate

Supplementary Table 1: Patient cohort and indications for n=66 fetal MRIs. PAE = prenatal alcohol exposure; BDE = binge drinking episodes. * Two patients had repeated fetal MRIs during pregnancy.

Fetal MRI finding	Abnormal	Unremarkable
Brain	7	14
Delayed opercularization	1	
Ventricular asymmetry	4	
Ventriculomegaly	2	
Microcephaly	1	
Plexus and frontal horn cysts	1	
Placenta	4	17
Hemorrhage	1	
Infarction	1	
Signs of venous congestion	2	

Supplementary Table 2: Prenatal MRI of fetuses with prenatal alcohol exposoure (PAE, n=21). In the PAE cohort, abnormalities of the brain (n=7, 33.0%) and placenta (n=4, 19.0%) were found.
